# Characterizing Structural Transitions Using Localized Free Energy Landscape Analysis

**DOI:** 10.1371/journal.pone.0005525

**Published:** 2009-05-13

**Authors:** Nilesh K. Banavali, Alexander D. MacKerell

**Affiliations:** 1 Laboratory of Computational and Structural Biology, Division of Genetics, Wadsworth Center, New York State Department of Health, Albany, New York, United States of America; 2 Department of Biomedical Sciences, The State University of New York at Albany, Albany, New York, United States of America; 3 Department of Pharmaceutical Sciences, School of Pharmacy, University of Maryland, Baltimore, Maryland, United States of America; Vanderbilt University, United States of America

## Abstract

**Background:**

Structural changes in molecules are frequently observed during biological processes like replication, transcription and translation. These structural changes can usually be traced to specific distortions in the backbones of the macromolecules involved. Quantitative energetic characterization of such distortions can greatly advance the atomic-level understanding of the dynamic character of these biological processes.

**Methodology/Principal Findings:**

Molecular dynamics simulations combined with a variation of the Weighted Histogram Analysis Method for potential of mean force determination are applied to characterize localized structural changes for the test case of cytosine (underlined) base flipping in a GTCAGCGCATGG DNA duplex. Free energy landscapes for backbone torsion and sugar pucker degrees of freedom in the DNA are used to understand their behavior in response to the base flipping perturbation. By simplifying the base flipping structural change into a two-state model, a free energy difference of upto 14 kcal/mol can be attributed to the flipped state relative to the stacked Watson-Crick base paired state. This two-state classification allows precise evaluation of the effect of base flipping on local backbone degrees of freedom.

**Conclusions/Significance:**

The calculated free energy landscapes of individual backbone and sugar degrees of freedom expectedly show the greatest change in the vicinity of the flipping base itself, but specific delocalized effects can be discerned upto four nucleotide positions away in both 5′ and 3′ directions. Free energy landscape analysis thus provides a quantitative method to pinpoint the determinants of structural change on the atomic scale and also delineate the extent of propagation of the perturbation along the molecule. In addition to nucleic acids, this methodology is anticipated to be useful for studying conformational changes in all macromolecules, including carbohydrates, lipids, and proteins.

## Introduction

Biological macromolecules and their complexes often undergo large structural changes during their functional cycles [1], [2]. Such structural changes can be distributed throughout many regions of the molecules [3], but they are usually localized to specific regions that transform internally [4], while other regions maintain their internal structure and move almost as rigid bodies. Irrespective of which category the structural changes belong to, a quantitative method that could identify and characterize the energetics of structural changes at the precision level of well-defined local degrees of freedom is highly desirable. Many experimental methods such as X-ray crystallography [5] or Nuclear Magnetic Resonance (NMR) Spectroscopy [6] can identify the atomic details of stable states involved in the structural changes. Biochemical assays of enzymatic function [7] or Fluorescence Resonance Energy Transfer (FRET) experiments [8] can characterize the kinetics and thermodynamics of the structural changes. However, it is typically difficult to experimentally obtain atomic structural details associated with the energetics of macromolecular structural change. Free energy determination methods using Molecular Dynamics (MD) simulations with empirical force fields [9]–[11] provide a framework to connect the structural and biochemical studies by determining free energy landscapes of global structural changes and the associated effective free energy landscapes of any local degree of freedom.

In the present study, single base flipping from a DNA double helix is used as a test case to point out how a structural change can be precisely characterized using free energy landscape analysis. Single base flipping out of a DNA duplex, without affecting the overall B-form duplex structure, was first demonstrated by the X-ray crystal structure of DNA bound to the *HhaI* cytosine-5-methyltransferase (M.*Hha*I) [12]. Subsequent biochemical and structural studies [13], [14] have shown base flipping to be a common structural mechanism employed by a variety of DNA repair and modification enzymes. The energetics of a related (but more limited) process of transient base pair opening in solvated DNA had been previously characterized using imino-proton exchange [15], [16] but the more extensive structural change associated with single base flipping had not been anticipated prior to the structural studies of M.*Hha*I. To carry out our MD simulations, we chose the well-studied DNA *HhaI* recognition sequence CCATGCGCTGAC as the test case, with the underlined cytosine base undergoing flipping. The single base flipping was studied using umbrella sampling [9] with restraints along a pseudodihedral reaction coordinate [17] that we had previously developed and applied in a number of studies of base flipping [18]–[22].

Other studies have probed the energetic characteristics of localized backbone degrees of freedom individually by using umbrella sampling [23] or unrestrained [24] MD simulations. These studies describe the correlated behavior of α-γ [23] or ε-ζ [24] torsions in specific base steps in the helical state of a DNA duplex. The present study goes further by probing the behavior of all localized torsions simultaneously in the background of the overall structural change involved in single base flipping as described before [25]. A two-state classification of the overall structural change allows identification of its effects on the smaller scale localized structural changes that either accompany it or drive it. This free energy analysis offers a detailed structural and energetic picture of a molecular conformational change.

## Results

### Two-state classification of the Watson-Crick stacked and high energy flipped states

**Figure 1:**
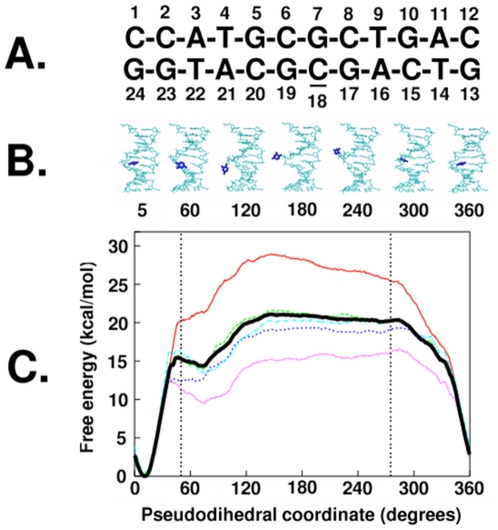
Free energy profile of the overall structural change during cytosine base flipping. A. Numbered sequence of the dodecamer used in the present study, underlined cytosine18 is the flipping base; B. Structures averaged over the last 2 ps of sampling in windows with pseudodihedral coordinates corresponding to labels, flipping cytosine18 shown in blue, rest of the DNA shown in cyan, harmonically constrained end basepairs are not shown; C. The Potential of Mean Force of Cytosine18 Base flipping along the periodic center-of-mass pseudodihedral reaction coordinate, Bold black line: entire 400 ps of sampling, red line: first 80 ps increment, dotted green line: second 80 ps increment, dotted blue line: third 80 ps increment, dotted pink line: fourth 80 ps increment, dotted cyan line: fifth 80 ps increment.

Base flipping is a structural change where an individual base moves out of its Watson-Crick (WC) base paired state, through either the minor or major groove, to a fully exposed state outside the double helix. Intermediate structures in the base flipping of the target cytosine (underlined in Figure 1A) are shown in Figure 1B with the associated free energy profiles or potentials of mean force (PMF) shown in Figure 1C. The free energy profile of the overall base flipping is similar to previously published PMFs [17]–[22] and shows the WC state in a deep energy well near 10° in the pseudodihedral coordinate with large barriers to flipping on either side (via the minor or major grooves) and a relatively flat landscape for the flipped states from 50° to 275°. The depth of the energy well for the WC paired state emphasizes the inherent stability of the DNA duplex and the energy required for a base to flip out of the double helix.

While there may be many subtleties in the base flipping process itself, on a coarser level, the nature of the one-dimensional free energy profile along the pseudodihedral restraint (Figure 1C) can be simplified into two states: the WC stacked state and the base flipped state. Table 1 indicates how dependent the calculated relative two-state free energy differences are based on the criteria used to classify the two states. If the sampling was divided into five subsets and different values of the one-dimensional pseudodihedral coordinate were used to classify the flipped and WC stacked states, the relative free energy differences vary from 6.2 kcal/mol to 20.9 kcal/mol. The details of how these values are obtained are given in the methods section. This variation was also apparent in the differences between the free energy profiles shown in Figure 1C.

**Table 1:** The two state free energy difference between stacked and flipped states for different ranges of the pseudodihedral coordinate used to define the flipped state and different sampling intervals.

Sampling	25°–345°	35°–335°	45°–325°	55°–315°	65°–305°	50°–275°
0–80 ps	7.2	14	18.2	19.7	20.9	20
80–160 ps	7.2	13	13.5	13.5	13.7	13.5
160–240 ps	7.2	11.4	11.7	11.9	12.2	11.8
240–320 ps	6.2	9.1	9.1	9.2	9.2	9.2
320–400 ps	7.9	12.8	12.8	12.8	12.8	12.8
0–400 ps	7.1	13.3	13.8	13.9	14.1	13.9

Energy differences in kcal/mol, pseudodihedral range shown at top of each column.

Considering the entire sampling range, the classification criteria alone could vary the relative free energies from 7.1 kcal/mol to 13.9 kcal/mol. This energy difference provides a link between the experimental measurements and the structural states that are being measured. The qualitative nature of the free energy profile as reflected by the mean force along the pseudodihedral reaction coordinate directs the cytosine base towards the lowest energy stacked state at values below 50° and values above 275° in all sampling sets. The flipped state windows with pseudodihedral values between these two borders were therefore likely to be metastable. All subsequent analysis used these boundaries to classify the two states (the borders of the flipped state region are shown in Figure 1C as vertical dotted lines) and used the entire sampling range for both the stacked and flipped states. The flipped states hold potential free energy corresponding to the free energy difference between the stacked and flipped states. Previous structural analysis suggested that the stress accompanying a single base flipping out of the double helix was localized to backbone regions between the nearest neighbor partners [18]. The present two-state classification based analysis of energy landscapes can confirm whether this stress also subtly changes the behavior of remote regions of the molecule.

In experimental studies that quantified base pair opening using imino-proton exchange, it was the relative probabilities of the stacked and opened states that were probed [15], [16]. However, there is evidence that these experiments may not measure the larger base flipping structural change observed in the presence of M.*Hha*I. It has been previously suggested that minimal correlated opening between the paired bases may be required for imino-proton exchange to occur [26], only locally open states may be involved, and opening of purine bases as opposed to pyrimidine bases may dominate the NMR measurements [21]. Opening rate constants from imino proton exchange are in the 100 s^−1^ range [15], [16] while more recent experimental studies based on trapping of the flipped base in β-cyclodextrin yield rate constants in the 10^−3^ s^−1^ range. The 5 orders of magnitude difference between the rate constants obtained from the two experimental approaches corresponds to an energy barrier difference of up to 7 kcal/mol assuming that the pre-exponential term in transition state theory is the same for both processes. The present study indicates that this difference can be bridged by including marginally flipped conformations in the classification of the flipped states resulting in a smaller energy difference. Our results also suggest that the higher rate constants obtained from imino proton exchange might be associated with local opening or marginally flipped states while the lower rate constants obtained by trapping experiments might be associated with full flipping of the base out of the double helix.

### Conformational analysis using free energy landscapes

**Figure 2:**
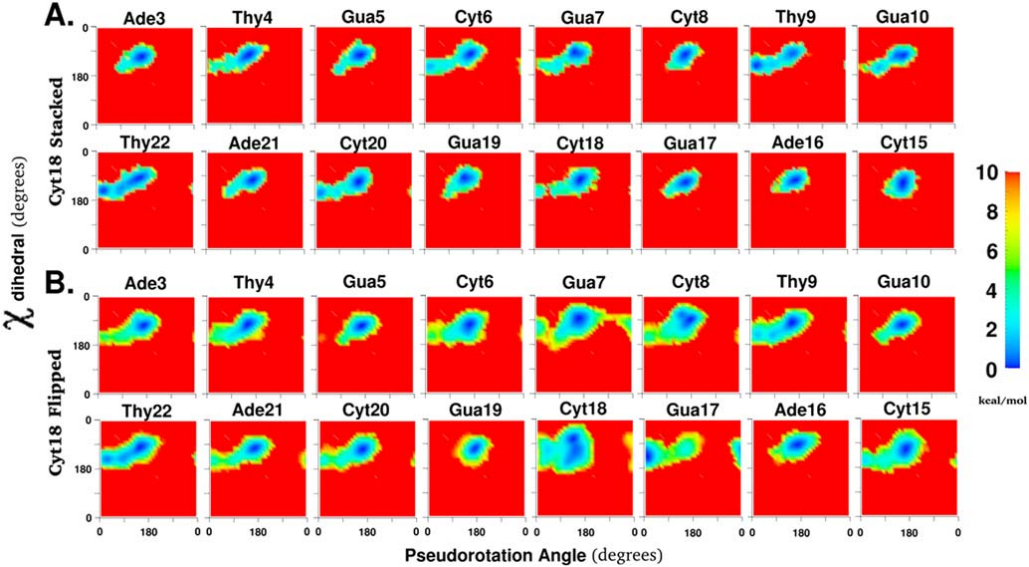
The two dimensional free energy landscapes of 8 central base positions along the pseudorotation angle and the glycosyl torsion, χ for A. all pseudodihedral coordinate windows and B. only pseudodihedral coordinate windows classified as flipped state windows as shown in Figure 1C. All torsion values are in degrees and energies are color coded in kcal/mol and truncated at 10 kcal/mol.

This study focuses on readily visualizing and quantifying the range of conformational space sampled by local degrees of freedom during a structural change in the macromolecule; which in the present case is the flipping of a base out of a DNA duplex. This was performed by calculating the unbiased effective free energy landscapes of the local degrees of freedom, as described below in the Materials and Methods. For example, all panels in figure 2 show the 2D free energy landscape of the sugar pucker vs χ degrees of freedom for specific base positions. The color coding for the conformations goes from blue to green to yellow through to red in the direction of increasing energies, with red representing the forbidden conformations. Throughout this study, the forbidden regions correspond to regions with relative energies of 10 kcal/mol or greater above the minimum for each 2D free energy landscape (see methods for justification of the value of 10 kcal/mol for truncation). The extent of convergence of calculated free energy landscapes is difficult to guarantee. Agreement with experimental results for the restrained degree of freedom could indicate that calculated free energy landscapes are representative of the experimental regimen. This issue of convergence is discussed in greater detail in the supporting information.

### Base and sugar degrees of freedom

The standard nomenclature of backbone torsions in DNA classifies them as α, β, γ, χ, ε, and ζ [27]. The conformation of the furanose sugar ring can be condensed into two combined degrees of freedom called the pseudorotation angle and pseudorotation amplitude [28]. The six aforementioned torsions and the pseudorotation angle substantially describe the conformational state of each individual nucleotide, in contrast to other geometric criteria used to describe the relationship between nucleotide and base-pair positions or the helical state of the DNA oligonucleotide [29]. Since the difference between individual nucleotide positions is the identity of the base attached to the sugar moiety, the correlation between pseudorotation angle and the glycosyl torsion χ is a localized conformational relationship of special interest in describing base composition and sequence dependent structural changes of the oligonucleotide.

Figure 2 shows the free energy landscape of motion along these two degrees of freedom for all 8 central base positions in the oligonucleotide for both the stacked state (Fig. 2A) and the flipped states (Fig. 2B). In the stacked state, all nucleotide positions, except Thy9, preferred the C2′-endo sugar pucker (P value around 180°) and the corresponding χ minimum around 260° (Table S1 of the Supplementary material). Interestingly, there were some clear differences between the behavior of the same base in different sequence contexts. For example, the order of relative preference for C2′-endo sugar pucker over C3′-endo sugar pucker was Thy4≫Thy22>Thy9. The sequence context beyond immediate neighbors also seemed to play a role since Thy4 and Thy22 show consistently different preferences in both stacked and flipped states even while having the same adjacent bases in the sequence (5′-Ade and 3′-Gua). However, the differences may also result from a lack of convergence of the sampling of conformational space. The lowest energy state minima and the alternate minima seen to be accessible for certain positions give a succinct description of the ground state dynamics of this particular DNA sequence in aqueous solution for the sugar pucker and χ torsion degrees of freedom.

In base positions that show transitions between C3′-endo and C2′-endo sugar puckers, the O4′-endo sugar pucker appears to be a metastable intermediate. The surfaces reveal two barriers separating the O4′-endo conformation from the C3′-endo and the C2′-endo conformation. One barrier between the C3′-endo and O4′-endo conformations requires only a change in the sugar pucker, while the second barrier between the O4′-endo conformation and the C2′-endo conformation requires correlated changes in both sugar pucker and χ torsions. The ability of the present free energy analysis to readily identify these paths represents an important attribute of the method.

When the flipped state was considered separately from the stacked state (Fig. 2B), the minima in these two local degrees of freedom shifted significantly for some base positions. The most substantial shifts occurred for Thy9 and Gua17, which inverted their preferences from C3′-endo and C2′-endo sugar puckers to C2′-endo and C3′-endo sugar puckers, respectively. In addition, the range of 2D conformational space sampled increased for all base positions studied. This broadening of the valley regions of the free energy landscape made more regions of local conformational space accessible for the flipped state. There were also other clear shifts for the higher energy regions in Ade3 (C3′-endo pucker more favorable), Cyt8 (C3′-endo pucker more favorable), Gua10 (C3′-endo pucker less favorable), Cyt15 (C3′-endo pucker more favorable), Cyt20 (C3′-endo pucker less favorable), and Ade21 (C3′-endo pucker more favorable). The perturbation caused by the overall structural change of Cyt18 flipping is clearly not restricted to the adjacent base pairs alone.

However, for the flipped state, the greatest perturbation occurred in the vicinity of the flipping base. The sugar pucker and χ torsions for the flipping Cyt18 sampled almost 25% of the total 2D conformational space available. The χ torsion was especially variable around the minimum of about 165°. This greatly increased flexibility is suggested to be associated with removal from the immediate constraints of the double helix. The orphan Gua7 base position also showed a remarkable characteristic: the so-called “west” barrier to sugar pucker change (*i.e.* pseudorotation angles about 270°), which was indicated to be extremely disfavored according to vacuum *ab initio* studies [30], was about 8 kcal/mol and comparable to the “east” barrier (*i.e.* pseudorotation angles about 90°). The Gua7 sugar could thus choose either barrier to invert sugar puckers when Cyt18 was flipped. Specific environmental conditions (in this case: no pairing with Cyt18 while base stacking is maintained) can change the conformational behavior of a specific nucleotide position in unexpected ways. Since protein binding and altered neighboring sequences both change the environment of individual DNA bases, the ability to quantify changes in conformational preferences of individual base positions due to environmental influences is likely to be very useful in understanding DNA-protein interactions and DNA sequence dependence.

### BI/BII equilibrium

**Figure 3:**
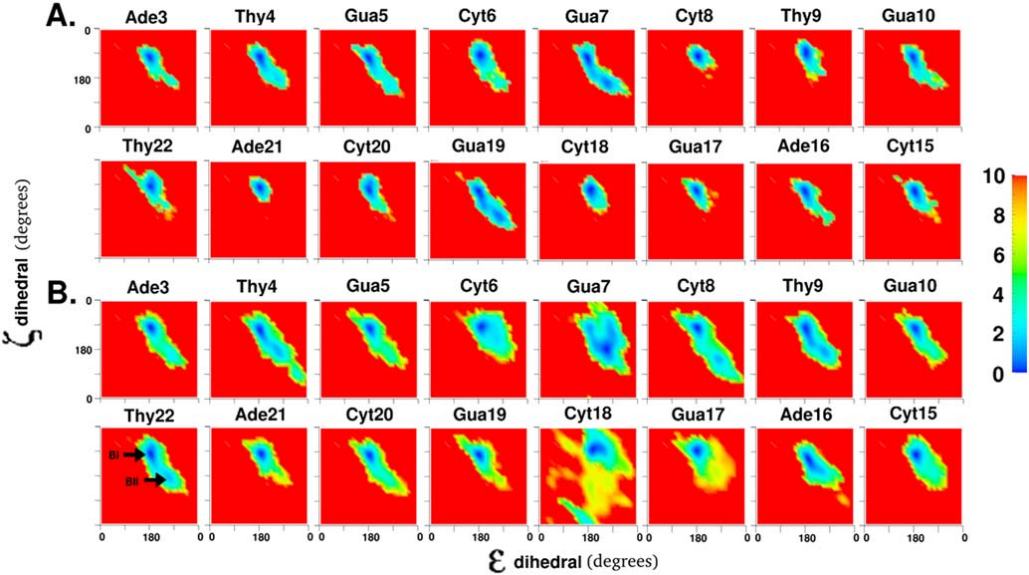
The two dimensional free energy landscapes of 8 central base positions showing the BI/BII equilibrium along the ε torsion and the ζ torsion for A. all pseudodihedral coordinate windows, B. only pseudodihedral coordinate windows classified as flipped state windows as shown in Figure 1C. Representative locations of the BI and BII form minima indicated by arrows and labels for the Thy22 position. All torsion values are in degrees and energies are color coded in kcal/mol and truncated at 10 kcal/mol.

The ε and ζ torsions in DNA populate two correlated minima called the BI and BII forms [31]. The BI form is defined as ε in the range 130°–210° and ζ in the range 235°–295° and the BII form is defined as ε in the range 210°–300° and ζ in the range 150°–210° [31]. The free energy landscapes of the ε and ζ torsions for the 8 central base pairs are shown in Figure 3. In the lower energy stacked state (Figure 3A), base positions Cyt8, Gua17, Cyt18, and Ade21 appear virtually locked in their BI form, while Gua7 and Gua19 show the greatest tendency to populate both the BI and BII forms.

In the flipped state, the broadening effect seen in χ and pseudorotation angle degrees of freedom was also present for all 16 central base positions in the ε and ζ torsional space (Figure 3B). The BII form became less favorable in base positions Gua5 and Gua19 as compared to the stacked state. In Thy4, an additional region of conformational space with ε around 300° and ζ around 60° became accessible through the BII form. The BII form became the lowest energy minimum for the orphan Gua7 base position with two low energy paths connecting it to the location of the original BI form and to another minimum at ε around 270° and ζ around 270°.

Cyt18 showed so much enlargement of conformational space sampled that almost 50% of the total available 2D conformational space was accessed. Unusual regions sampled included: ε around 90° and ζ around 90°, ε around 90° and ζ around 300°, ε around 180° and ζ around 0°, and ε around 180° and ζ around 180°. Clearly, the flipped state of Cyt18 acquired a significant amount of flexibility in their ε and ζ degrees of freedom. Given that these degrees of freedom are correlated with the sugar pucker, it could be argued that the altered behavior of oligonucleotides with conformationally locked sugar substitutions [32]–[34] is caused by a complex combination of factors including alterations in the sampling of external torsional degrees of freedom in addition to changes in the constrained sugar conformation alone.

### Sugar and phosphodiester backbone

**Figure 4:**
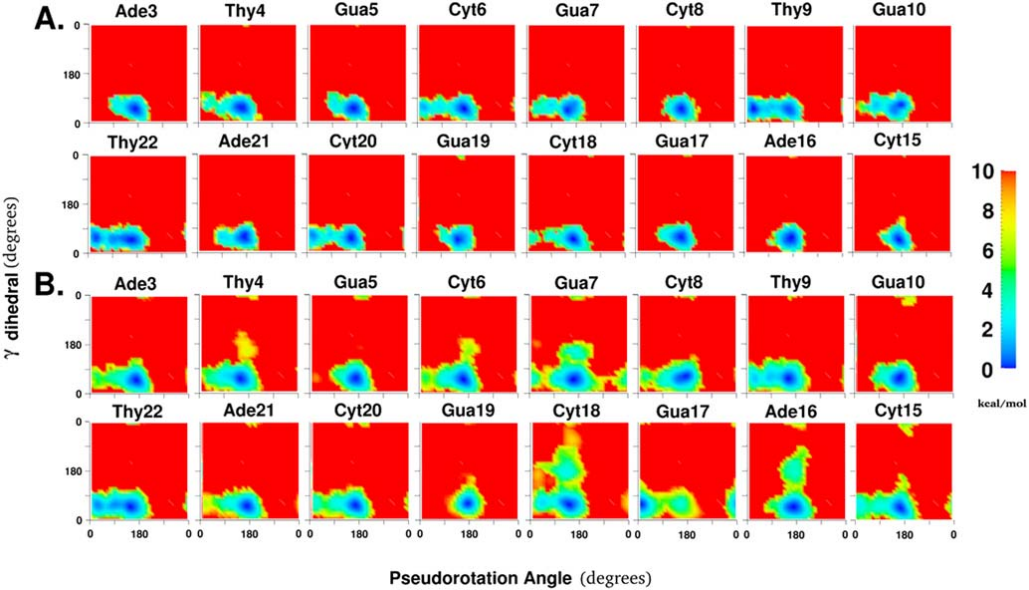
The two dimensional free energy landscapes of 8 central base positions along the γ torsion and the pseudorotation angle for A. all pseudodihedral coordinate windows, B. only pseudodihedral coordinate windows classified as flipped state windows as shown in Figure 1C. All torsion values are in degrees and energies are color coded in kcal/mol and truncated at 10 kcal/mol.

Among torsions that connect the sugar through the phosphodiester linkages to adjacent sugars, the γ torsion lies immediately to the 5′-side of each sugar, and the ε torsion lies immediately to its 3′-side. The correlated changes in the free energy landscapes of the γ torsion and the pseudorotation angle for the 8 central base pairs are shown in Figure 4. One clear characteristic that agrees with crystallographic observations on B-form DNA structures [35] is that the γ torsion prefers the *gauche−* minimum around 55° in all nucleotide positions. The γ torsion and the pseudorotation angle can sample regions within a diameter of approximately 90° around the primary minimum in the 2D torsional space. As seen above with the χ degree of freedom, the sampling range is greater for positions that show the possibility of exchange between C3′-endo and C2′-endo sugar puckers such as Thy4, Cyt6, Gua7, Thy9, Gua10, Cyt18, Cyt20, and Thy22, but only in the pseudorotation angle dimension, with γ maintaining its original minimum.

When only the flipped state is considered (Figure 4B), the general broadening effect and sugar pucker preference changes accompanying flipping seen above are observed in these degrees of freedom as well. In addition, the *trans* state of the γ torsion is now accessible in the nucleotide positions of Thy4, Cyt6, Gua7, Ade16, and the flipping Cyt18. The similarities between Ade16 and the flipping Cyt18 are especially notable since the intervening Gua17 base showed no change in preference for the γ torsion and a complete shift to the C3′-endo sugar pucker. This is consistent with our previous observations [17] that the flipping base can interact with regions of the DNA duplex beyond its immediately adjacent base pairs while it undergoes base flipping through either the minor or major grooves. The greatest broadening effect for the free energy landscape again occurs in the vicinity of the flipping site. Cyt18 itself shows not only a clear metastable minimum for the *trans* state of the γ torsion but also greater accessibility of the *gauche+* state with the C2′-endo sugar pucker, and the *trans* state with the C3′-endo sugar pucker. These observations do not contradict structural data from experiment, since the flipping base is free of the constraints of the canonical double helix and probably resembles more the behavior of individual nucleotides where these conformations are not unusual [36]. In crystal structures of this sequence bound to the *HhaI* methyltransferase, the *trans* state of the γ torsion of the flipping position was consistently observed, irrespective of the presence or identity of the flipping base [32]. This again illustrates how alternative conformations of local torsions stabilized through modifications of the environment contribute to larger scale structural changes such as base flipping.

**Figure 5:**
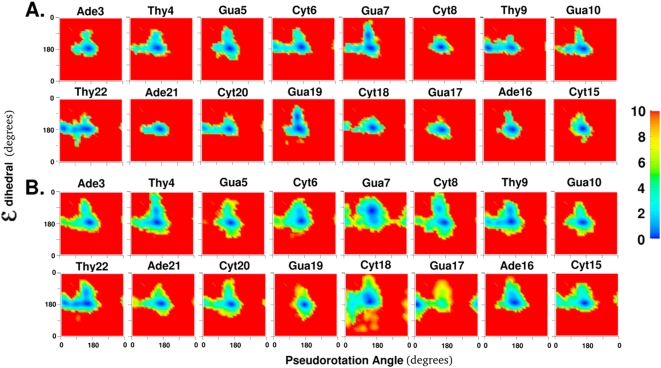
The two dimensional free energy landscapes of 8 central base positions along the pseudorotation angle and the ε torsion for A. all pseudodihedral coordinate windows, B. only pseudodihedral coordinate windows classified as flipped state windows as shown in Figure 1C. All torsion values are in degrees and energies are color coded in kcal/mol and truncated at 10 kcal/mol.

The free energy landscapes of the pseudorotation angle and ε torsions for the 8 central base pairs are shown in Figure 5. In the stacked state (Figure 5A), the pseudorotation angle preferences are as described above, but the correlated motion in the ε torsion indicates that the BI/BII equilibrium mostly accompanies the C2′-endo sugar pucker and consequently the B-form of DNA. Most base positions show accessibility to ε torsion values (around 260°) usually found in the BII form and not in the BI form [37]. Some base positions (Cyt8, Gua17, Cyt18, and Ade21, and to a lesser extent Thy9, Cyt15, Cyt20, and Thy22) did not show such accessibility. Notably, ε torsion values around 260° were not found to accompany pseudorotation angles below 90° corresponding to C3′-endo sugar puckers. This is reflected in the inverted L-shaped 2D free energy landscapes for nucleotide positions that show change in both sugar pucker and ε torsion minima (e.g. Thy4 and Gua7). If these higher ε torsion values were to accompany C3′-endo sugar puckers, these energy landscapes are expected to be U-shaped.

In the flipped states (Figure 5B), the familiar broadening of the free energy landscape valleys also occurs in these two dimensions, but with varying trends for individual positions. While most nucleotides showed increased accessibility to ε torsion values in the range of 260°, some base positions (Gua5 and Gua19) actually showed decreased accessibility. These higher ε torsion values also did not occur with the C3′-endo sugar pucker even in the flipped states. The exception to this rule was the orphan Gua7 base position, which not only shifted its lowest energy state to these higher ε values corresponding to the BII form (Table S1 of the Supplementary material), but also allowed them to accompany its C3′-endo sugar pucker conformations, resulting in a roughly U-shaped landscape. Cyt18 also had much greater flexibility when it flipped out of the double helix, especially noticeable in its access of the unusual state where its sugar is in the C3′-endo conformation and its ε torsion value is around 100°.

### Helicoidal parameters

DNA conformation could also be understood in the context of base step or helicoidal parameters [38]. While we have focused our analysis on dihedral degrees of freedom, the present method may also be applied to analyze these base step and helicoidal parameters. As an example, the approach has been applied to twist and roll base step parameters and the results are included in the supporting material (see supplementary material figure S2 and text S1 for more details).

## Discussion

Umbrella sampling MD simulations typically condense the complex multidimensional free energy landscape of specific conformational changes in macromolecules into one or two reaction coordinate dimensions [17], [39]. Depending on the choice of reaction coordinate, the free energy profiles obtained provide uncomplicated insights into the properties of the specific processes studied. However, the conformational changes themselves are a complex combination of individual degrees of freedom at the atomic scale, amongst which, internal degrees like atomic bond lengths and angles are relatively rigid, while rotations around bonds are typically more flexible, often making important contributions to the studied conformational change. Changes in non-bonded interactions with other parts of the macromolecule and the solvent environment are also important in the conformational changes. The present study presents a method to extract the complex localized conformational characteristics that contribute to larger conformational changes through the calculation of free energy landscapes of local degrees of freedom. A systematic analysis of the contribution of local torsions in single base flipping out of a DNA duplex was used as a test case.

Delving into the complexities associated with conformational changes becomes necessary because the simplification into a single reaction coordinate in umbrella sampling typically does not allow for mechanistic explanations for larger structural changes being observed to be derived. When certain single base mutations result in profound changes in conformational behavior or molecular recognition [40], [41], a convincing mechanistic explanation is often elusive. When a very localized change results in a change in behavior for the entire macromolecule, the reason behind the change presumably needs to be tracked down to its local origins. In this case, the wealth of information in MD simulations is an advantage because it allows quantification of the complex free energy landscape with respect to the local degrees of freedom of interest. In the present study, local features contributing to the structural change of base flipping on a DNA oligonucleotide are clarified by determining the local torsional free energy landscapes. The higher free energy (>10 kcal/mol) conferred on the double helix due to base flipping is partially stored by the flipped state as unfavorable torsions in the immediate vicinity of the flipping base, but also as unfavorable torsions in remote parts of the oligonucleotide. This dispersal of stress is not uniform, with certain remote nucleotides and certain torsions within the duplex showing greater susceptibility to assume unfavorable conformations. This is presumably related to the long-range relationships between the backbone moieties or direct interactions with the flipping base itself, as previously observed [17]. Some of the more prominent examples are the γ torsion in Ade16, the pseudorotation angle in Cyt15, and the BI/BII equilibrium in Thy22. Free energy landscape analysis also indicates unusual sequence dependent conformational properties of certain nucleotides unrelated to Cyt18 flipping. For example, Thy4, Thy9 and Thy22 show larger stabilization of the C3′-endo sugar pucker, while Cyt8 and Ade21 show much lower stability of the BII form even when Cyt18 is in its stacked state.

In future studies, the criteria for subdivision of higher energy states can be branched further to obtain more detailed understanding of specific local conformational changes contributing to different aspects of the overall structural change. The differences in free energy landscapes of local degrees of freedom between the two global states can also be quantified more precisely by overlap integral calculations [42]. The effect of environment (water and ions) is included in the localized free energy landscapes, but a more complete mechanistic decomposition of this effect is desirable. Since the free energy landscape can be extracted for any degree of freedom, free energy landscapes of order parameters describing water structure or ion positions can also be determined by the present method. The ability to energetically analyze macromolecular behavior in such detail also provides a good calibration tool for force field development by helping to relatively quickly determine how the free energy of alternate conformations is modulated by specific changes in force field parameters. Differences in free energy landscapes of local order parameters for the same nucleotide in varying sequence contexts will provide a quantitative measure of sequence dependence. This potential suggests that by routinely applying the present analysis to different nucleic acid sequences undergoing various structural changes, it may be possible to achieve a detailed and robust understanding of the relationship between base composition, base sequence and structural heterogeneity in nucleic acids. Finally, it should be reiterated that free energy landscape analysis is general and can be easily applied to any macromolecule to get an atomic-scale characterization of its biological function.

## Materials and Methods

The sequence used in this study, CCATGCGCTGAC, is the recognition sequence for the *HhaI* cytosine-5-methyl transferase, where the underlined C represents the flipping base (Figure 1). It was chosen to allow comparison to previous studies [17], [43], [44] and for its biological relevance. The preparation of the system used in this study was described before [17] and is only briefly summarized here. The program CHARMM [45] and the CHARMM27 nucleic acid force field [46], [47] with the CHARMM modified TIP3P water model [48], and sodium parameters from Beglov and Roux [49] were used for all calculations. The minimized B-form dodecamer was solvated in a 61 Å×61 Å×61 Å cube with enough randomly distributed sodium ions to electrostatically neutralize the system. Long-range electrostatic interactions were treated using the Particle Mesh Ewald (PME) approach [50] with a B-spline order of 4 and a Fast Fourier Transform grid of one point per Å and a real-space Gaussian width kappa of 0.3 Å^−1^. Real space and Lennard-Jones (LJ) interaction cutoffs of 8 Å were used with non-bond interaction lists maintained and heuristically updated out to 16 Å. The entire system was minimized and the solvent environment was equilibrated for 20 ps using a constant volume, isothermal (NVT) ensemble MD simulation with the DNA non-hydrogen atoms kept harmonically restrained. The system was equilibrated for a further 0.5 ns of constant pressure, isothermal (NPT) [51] dynamics without restraints.

The procedure for calculation of the free energy of base flipping using umbrella sampling with the previously described pseudodihedral coordinate [17] is slightly modified in this study. Harmonic restraints with force constants of 2 kcal/mol were applied to non-hydrogen atoms of 5′ and 3′-end base pairs to prevent the end base-pair fraying observed in the previous study. This restraint also prevented translation and rotation of the DNA within the periodic simulation box. For the umbrella sampling, a periodic pseudodihedral harmonic restraint was used:
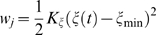
(1)where *w_j_* is the restraint energy, *K_ξ_* is the force constant in kcal/mol/radian^2^, *ξ(t)* is the value of the pseudodihedral ξ at time *t*, and *ξ_min_* is the value to which the pseudodihedral is restrained. To impose periodicity in the restrained pseudodihedral, we use the following function:
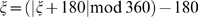
(2)All 72 windows spanning the periodic range (5° intervals for a total range of 360°) of the pseudodihedral coordinate describing base flipping were generated by using the above restraint with a large force constant of 10000 kcal/mol/radian^2^ and 20 ps of NPT MD simulation to equilibrate each window before using the final coordinates as starting coordinates for the next window. This is in contrast with the very fast flipping used in the original study to generate initial coordinates and presumably allowed the system to absorb the flipping change more gradually. These initial windows were then used as starting points for umbrella sampling MD simulations with the pseudodihedral restraint. The final system in each window, consisting of 21306 atoms including 6842 waters and 22 sodium ions, was then allowed to evolve with a pseudodihedral restraint of 1000 kcal/mol/radian^2^ for a further 0.4 ns of NPT MD simulations, all of which were saved at each step of the dynamics and used for subsequent analysis. The total simulation time for all windows combined was 30 ns. The periodic WHAM algorithm [39], [52] was used to calculate the potential of mean force (PMF) along the one-dimensional pseudodihedral reaction coordinate from the time series of this variable saved at every integration step of the 0.4 ns production dynamics. This highest frequency of sampling was used to prevent capturing any brief transition states, however, it is possible to reduce the frequency of sampling to once every 50 steps without a substantial change in the energy profiles (see supplementary material figure S1 and text S1 for more details)

As explained in a previous study on global structural change in DNA [25], to calculate free energy landscapes of local degrees of freedom, first the biased probability distribution, *<ρ_bias_(ξ_1_,ξ_2_)>* for the two variables: *ξ_1_* = restrained pseudodihedral, *ξ_2_* = any unrestrained localized variable such as a backbone torsion, was calculated from the time series of these two variables saved at each dynamics step in all umbrella sampling windows. The unbiased probability distribution *<ρ(ξ_1_,ξ_2_)>* was then calculated from *<ρ_bias_(ξ_1_,ξ_2_)>* using the Weighted Histogram Analysis Method (WHAM) equations [52]:
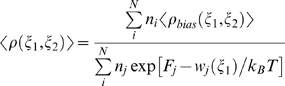
(3)

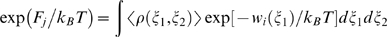
(4)where *N* is the number of windows, *n_i_* is the number of bins, *n_j_* is the number of windows, *F_j_* are the free energy constants for each window and *w_j_* is the biasing harmonic potential imposed along the pseudodihedral reaction coordinate shown in Eq. (1). The above WHAM equations minimize the overlap error between different windows that span the restrained pseudodihedral coordinate and corrects for the effect of the imposed artificial restraint. They therefore provide an accurate estimate of the continuous unrestrained free energy profile spanning the overall structural change along the pseudodihedral coordinate, inclusive of the local degrees of freedom whose free energy landscapes are being probed. A converged value of *<ρ(ξ_1_,ξ_2_)>* was obtained by iterating through Eq. (2) and Eq. (3) using a convergence criterion of 0.0001 in successive *F_j_* values. The unbiased density *<ρ(ξ_2_)>* for the unrestrained coordinate *ξ_2_* was obtained from:
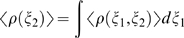
(5)and the free energy surface represented by *W*(*ξ_2_*) was obtained from:
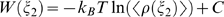
(6)where *C* is an arbitrary constant. It is important to note that *W*(*ξ_2_*) was calculated without enforcing sampling along the unrestrained dimension, *ξ_2_*, such that the entire range accessible to this dimension might not necessarily be explored. However, adequate sampling of the local, unrestrained degrees of freedom was assumed once convergence of the free energy profile along the original restrained pseudodihedral coordinate was attained (see supplementary material text S1 for an expanded discussion).

The two state free energy difference values shown in Table 1 were obtained by defining two specific ranges of the pseudodihedral windows as stacked and flipped, then integrating the Boltzmann factor exp[−*W(ξ_stacked_)/k_B_T*] or exp[−*W(ξ_flipped_)/k_B_T*] for those ranges in the pseudodihedral coordinate ξ, and finally getting the difference between the resulting free energy values *W(ξ_stacked_)* and *W(ξ_flipped_)* for the corresponding two state classification. Free energies of all localized degrees of freedom were truncated at a cutoff of 10 kcal/mol with all the windows along the pseudodihedral coordinate being used to calculate the free energy landscapes. This truncation level was used for three reasons: (a) it separates the WC stacked states (which are all below 10 kcal/mol) and flipped states (which are all above 10 kcal/mol); (b) a cutoff of 10 kcal/mol applied to only flipped states includes all flipped states (since all flipped states lie between 10 kcal/mol and 20 kcal/mol in overall free energy values); and (c) the probability of finding a specific conformation with a relative free energy of 10 kcal/mol is exponentially smaller (less than 10^−7^) compared to the lowest energy conformation. It should be noted that generating separate histograms for two states without using the present WHAM methodology would be sufficient for simulations where no restraint potential is used. However, it would yield incorrect results for simulations with a biasing restraint potential.

In the present work, the free energy landscape protocol was applied to two unrestrained dimensions, though the method is certainly extensible to three or more conformational degrees of freedom. A description of how the method can be extended to two unrestrained degrees of freedom is included in the supporting material. However, inclusion of additional degrees of freedom in the free energy landscapes might make the histograms very sparse in individual windows and convergence might be more difficult. All analysis of torsion angle related parameters was performed using either the FREEHELIX98 program [54] modified to read CHARMM trajectories or the 3DNA program [38]. Molecular pictures were produced using DINO (http://www.dino3d.org) and graphs were made using either gnuplot (http://www.gnuplot.org) or OPENDX (http://www.opendx.org) and compiled using GIMP (http://www.gimp.org) software.

## Supporting Information

Text S1 (0.04 MB DOC)

Table S1 (0.06 MB DOC)

Figure S1 (2.41 MB TIF)

Figure S2 (0.70 MB TIF)
