# Supplementary material for: Characterizing Structural Transitions Using Localized Free Energy Landscape Analysis
Source: PLoS One. 2009 May 13;4(5):e5525. doi: 10.1371/journal.pone.0005525 (PMC2678196; doi:10.1371/journal.pone.0005525)
Supplement: Text S1 — (0.04 MB DOC) [file pone.0005525.s001.doc]

**Supplementary Material: Characterizing Structural Transitions Using Localized Free Energy Landscape Analysis**

**Nilesh K. Banavali and Alexander D. MacKerell Jr.**

**PLOS ONE**

**Two unrestrained local degrees of freedom**

The biased probability distribution, *<ρ_bias_(ξ_1_,ξ_2_,ξ_3_)>* for the three variables, where *ξ_1_* is the restrained pseudodihedral, and *ξ_2_* and *ξ_3_* are unrestrained variables can be calculated from the time series of these three variables in all umbrella sampling windows. The unbiased probability distribution *<ρ(ξ_1_,ξ_2_,ξ_3_)>* can be calculated from *<ρ_bias_(ξ_1_,ξ_2_,ξ_3_)>* by solving the WHAM equations given below iteratively to convergence:

Eq. (1)

Eq. (2)

Eq. (3)

Eq. (4)

where *N* is the total number of windows, *n_i_* is the number of bins, *n_j_* is the number of restraint windows, *F_j_* are the free energy constants for each restraint window, *w_j_* is the biasing harmonic potential imposed in each restraint window, *W (ξ_2_,ξ_3_)* is the free energy profile along two unrestrained local degrees of freedom *ξ_2_* and *ξ_3_*. This can be extended to *n* degrees of freedom *ξ_2 …_ ξ_n_*, but these multidimensional histograms are also likely to be very sparsely populated on average.

**Convergence of local conformational energy landscapes**

Since unrestrained degrees of freedom are not enforced to sample their entire conformational range, the possibility cannot be completely ruled out that certain slower processes orthogonal to the restraint potential may not be converged. This is a general caveat in all restraint potential based free energy estimation methods and is related to the choice for the restraint “reaction coordinate” and the process that is desired to be studied. In this particular case, such orthogonal processes might include fraying of the DNA ends, which could occur without any direct impact on the restrained pseudodihedral. The free energy landscape analysis that we advocate here can quantify such processes but their slower convergence poses a sampling problem common to most molecular dynamics simulations.

The convergence of the degrees of freedom that are not completely orthogonal to the restrained coordinate is more critical. These are the local variables that are “pushed” into higher energy regions and actively respond to the restraint potential. We expect that they should adjust more quickly against an enforced structural change because they would be seeking lower energy regions along a tilted (biased) energy gradient (not a flat diffusive gradient) and they require relatively modest atomic level structural changes to do so. The relaxation of these “unrestrained” coordinates is probably the cause of the initial variability in the calculated free energy profiles. In the case where these local degrees of freedom are seen to occupy states not seen in B-form DNA structures, their lack of change upon increased sampling could be used as a metric to judge their convergence. Two apparent problems with such tests of individual degrees of freedom could be: (1) convergence in one such unrestrained coordinate might not guarantee convergence in correlations between different unrestrained coordinates; (2) the possibility that further sampling may result in changes can never be excluded.

**Dependence on sampling frequency of pseudodihedral coordinate**

The highest possible frequency of sampling the pseudodihedral coordinate utilized in the present study (each dynamics step) is merely to avoid missing any transient intermediate states. In order to understand how much less frequently one could save the reaction coordinate (or the molecular trajectories), we varied the sampling frequency and recalculated the free energy profiles of the overall base flipping. As shown in Supplementary Figure S1, one can obtain very similar results by reducing the sampling frequency to once every 50 steps, (thereby reducing disk space requirement by a factor of 50). Minor changes crop up when the sampling frequency is reduced further to every 100 or 200 steps, but reducing it further to once every 500 or 1000 steps causes the free energy profiles to show appreciable changes. Considering this factor is important because the error introduced due to reduced sampling frequency could be as high as 2 – 3 kcal/mol for higher energy regions.

**Analysis of base step parameters**

Even though we have primarily focused on the backbone torsions as the local variables to probe using the WHAM analysis during base flipping, it is indeed possible to probe any other geometrical variable in a similar fashion. Supplementary figure S2 shows free energy landscape analysis of the correlated changes of the twist and roll parameters for the central region of the oligonucleotide in all states. It is clear that the maximum distortion in these two parameters (sampled every 100 steps and analyzed using the program 3DNA) is localized in two base steps: the G-C base step (i.e. strand 1 gua7 and strand 2 cyt18) and the subsequent C-G base step (i.e. strand 1 cyt8 and strand 2 gua17). There is clearly a significant distortion from the normal distribution located between 20° to 60° in the twist parameter and -20° to 20° in the roll parameter for these base steps.

**Supplementary Figure Captions:**

**Figure S1:** Free energy profile of the overall structural change during cytosine base flipping for different sampling frequencies (from each step to every 1000 steps) of the pseudodihedral coordinate. Reduced sampling frequencies upto every 50 steps introduce insignificant changes, but changes become larger if the sampling frequency reduces beyond once very 500 steps. Thus it is possible to introduce unnecessary errors by sampling infrequently.

**Figure S2:** Localized free energy profiles for roll and twist base step parameters for central region of oligonucleotide for a sampling frequency of 100 steps. Distortion during base flipping is localized to mainly 2 base steps in these two parameters. The 3DNA program was not able to determine these parameters for flipped state pseudodihedral windows above 80° and below 290° for the G7-C18 base step due to the flipping of the C18 base.
