# Supplementary material for: Characterizing Structural Transitions Using Localized Free Energy Landscape Analysis
Source: PLoS One. 2009 May 13;4(5):e5525. doi: 10.1371/journal.pone.0005525 (PMC2678196; doi:10.1371/journal.pone.0005525)
Supplement: Table S1 — (0.06 MB DOC) [file pone.0005525.s002.doc]

**Table S1.** The coordinates of the lowest energy states in the 2D free energy landscapes along pseudorotation angle (P) and χ, γ and P, P and ε, and ε and ζ for the 8 central base positions considering either all states (no subscript) or only flipped states (indicated by the subscript F) along the pseudodihedral coordinate.

| Base | (P,χ) | (P, χ)_F_ | (γ, P) | (γ, P)_F_ | (P, ε) | (P, ε)_F_ | (ε, ζ) | (ε, ζ)_F_ |
| --- | --- | --- | --- | --- | --- | --- | --- | --- |
| Ade3 | 165,255 | 175,255 | 55,165 | 55,165 | 165,185 | 165,185 | 185,255 | 185,255 |
| Thy4 | 155,255 | 145,255 | 55,155 | 55,145 | 165,185 | 155,185 | 185,255 | 185,255 |
| Gua5 | 165,255 | 165,245 | 55,165 | 55,165 | 165,185 | 165,185 | 185,255 | 185,255 |
| Cyt6 | 165,255 | 165,255 | 55,165 | 55,165 | 165,195 | 165,195 | 195,265 | 195,265 |
| Gua7 | 145,265 | 165,275 | 45,145 | 45,155 | 165,185 | 155,255 | 185,255 | 245,185 |
| Cyt8 | 165,255 | 175,275 | 55,165 | 65,175 | 165,195 | 175,185 | 195,255 | 185,255 |
| Thy9 | 25,215 | 155,265 | 55, 25 | 55,155 | 25,185 | 155,185 | 185,275 | 185,255 |
| Gua10 | 175,255 | 165,255 | 65,175 | 45,165 | 175,185 | 165,185 | 185,255 | 185,255 |
| Cyt15 | 165,245 | 165,245 | 45,165 | 45,165 | 165,185 | 165,185 | 185,255 | 185,255 |
| Ade16 | 165,255 | 165,265 | 55,165 | 55,165 | 175,195 | 165,195 | 185,255 | 195,235 |
| Gua17 | 165,245 | 15,225 | 55,165 | 55, 15 | 165,185 | 15,185 | 185,255 | 185,275 |
| Cyt18 | 165,255 | 135,285 | 55,165 | 55,135 | 165,185 | 145,195 | 195,265 | 195,275 |
| Gua19 | 145,265 | 175,255 | 45,145 | 65,175 | 155,195 | 175,185 | 185,255 | 185,255 |
| Cyt20 | 155,245 | 165,255 | 55,155 | 55,165 | 165,185 | 165,185 | 185,255 | 185,255 |
| Ade21 | 175,255 | 165,255 | 55,165 | 55,165 | 175,185 | 165,185 | 185,265 | 185,265 |
| Thy22 | 165,265 | 145,255 | 55,155 | 45,145 | 155,185 | 155,185 | 185,265 | 185,265 |

All values are in degrees.
