# Supplementary figures and images for: Characterizing Structural Transitions Using Localized Free Energy Landscape Analysis
Source: PLoS One. 2009 May 13;4(5):e5525. doi: 10.1371/journal.pone.0005525 (PMC2678196; doi:10.1371/journal.pone.0005525)

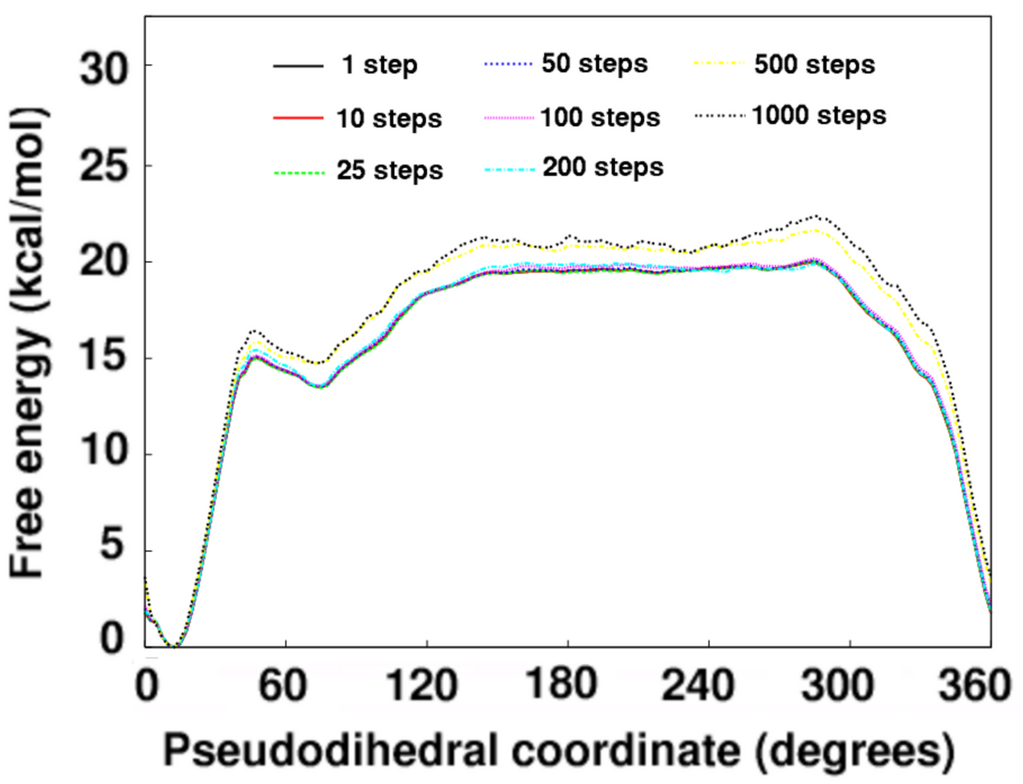

Supplement: Figure S1 — (2.41 MB TIF) [file pone.0005525.s003.tif]

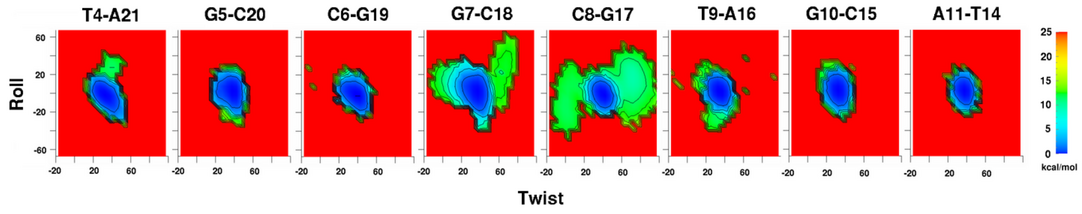

Supplement: Figure S2 — (0.70 MB TIF) [file pone.0005525.s004.tif]
